# Male Sexual Dysfunction and Infertility in Spinal Cord Injury Patients: State-of-the-Art and Future Perspectives

**DOI:** 10.3390/jpm12060873

**Published:** 2022-05-26

**Authors:** Francesco Di Bello, Massimiliano Creta, Luigi Napolitano, Gianluigi Califano, Francesco Passaro, Simone Morra, Angelo di Giovanni, Giovanni Maria Fusco, Luigi Cirillo, Marco Abate, Vincenzo Morgera, Gianluigi Cacace, Luigi De Luca, Gianluca Spena, Claudia Collà Ruvolo, Francesco Paolo Calace, Celeste Manfredi, Roberto La Rocca, Giuseppe Celentano, Carmine Turco, Marco Capece, Carlo D’Alterio, Alessandro Giordano, Ernesto di Mauro, Francesco Trama, Ugo Amicuzi, Davide Arcaniolo, Ferdinando Fusco, Nicola Longo

**Affiliations:** 1 Department of Neurosciences, Reproductive Sciences and Odontostomatology, Urology Unit, University of Naples “Federico II”, 80138 Napoli, Italy; fran.dibello12@gmail.com (F.D.B.); max.creta@gmail.com (M.C.); gianl.califano2@gmail.com (G.C.); francescopassaro1996@gmail.com (F.P.); simone.morra@unina.it (S.M.); angelodigiovanni92@gmail.com (A.d.G.); giom.fusco@gmail.com (G.M.F.); cirilloluigi22@gmail.com (L.C.); marcoabate5@gmail.com (M.A.); vincemorgera87@gmail.com (V.M.); cacace.gianlu@gmail.com (G.C.); luigideluca86@gmail.com (L.D.L.); spena.dr@gmail.com (G.S.); c.collaruvolo@gmail.com (C.C.R.); frap.calace@gmail.com (F.P.C.); robertolarocca87@gmail.com (R.L.R.); dr.giuseppecelentano@gmail.com (G.C.); car.turco87@gmail.com (C.T.); drmarcocapece@gmail.com (M.C.); cdalterio95@gmail.com (C.D.); alessandro.giordano24@gmail.com (A.G.); ernestodm9@gmail.com (E.d.M.); u.amicuzi@gmail.com (U.A.); nicola.longo@unina.it (N.L.); 2 Urology Unit, Department of Woman, Child and General and Specialized Surgery, University of Campania “Luigi Vanvitelli”, 80138 Naples, Italy; manfredi.celeste@gmail.com (C.M.); davide.arcaniolo@gmail.com (D.A.); ferdinando-fusco@libero.it (F.F.); 3 Department of Andrology and Urogynecological Clinic, University of Perugia, Santa Maria Terni Hospital, 05100 Terni, Italy; francescotrama@gmail.com

**Keywords:** andrology, spinal cord injury, male infertility, sexual dysfunction, erectile dysfunction, pathophysiology, personalized treatment

## Abstract

Spinal cord injury (SCI) is a relevant medical and social problem. According to the World Health Organization, the commonly estimated worldwide annual incidence of SCI is 40 to 80 cases per million population. After the SCI experience, most men present with sexual dysfunction (erectile dysfunction (ED) and ejaculatory dysfunction), fertility problems (such as impaired spermatogenesis, abnormalities in sperm viability, motility, and morphology), and systemic disorders such as genitourinary infection and endocrine imbalances. The best options available for managing the ejaculatory disorders in patients suffering from SCI are penile vibratory stimulation (PVS) and electroejaculation (EEJ). Furthermore, the treatment of ED in SCI patients consists of medical therapies including phosphodiesterase 5 inhibitors (PDE5i), intracavernosal injections (ICI), vacuum erection devices (VEDs), and surgical as penile prosthesis (PP). This review provides a snapshot of the current evidence for the mechanisms of sexual dysfunction and infertility in SCI patients, discusses the best management strategies for these conditions, and offers our perspective on the direction of future research.

## 1. Introduction

Spinal cord injury (SCI) is a not common injury, and in 70% of patients, SCI is concomitant with spinal cord trauma, increasing the rates of the complications that follow during both acute and extended recovery times [1,2]. There are several causes of SCI (Table 1), such as automobile crashes, falls, gunshot wounds, motorcycle crashes, diving incidents, medical/surgical complications (such as cancer, arthritis, inflammation of the spinal cord, or osteoporosis), and sport and recreation injuries (such as those from impact sports) [3]. According to the World Health Organization, the estimated worldwide annual incidence of SCI is 40 to 80 cases per million population [4,5]. Among those with SCI, there are young adult males in their reproductive years [6]. In this population, after SCI, several sexual consequences can occur, such as (ED and ejaculatory dysfunction), fertility problems (such as impaired spermatogenesis, abnormalities in sperm viability, motility, and morphology), and systemic disorders, such as genitourinary infection and endocrine imbalances [7,8,9,10,11,12]. ED represents the major concern in both complete and incomplete SCI patients, especially when associated with chronic disease [13,14,15,16]. Furthermore, the level and completeness of the SCI influence sexual functioning following the SCI [17]. As a result, sexual dysfunction and fertility management have become fundamental issues in this population in order to ensure their capacity to reproduce [18]. We performed a narrative review of the current literature to provide an overview of the mechanisms of sexual dysfunction and infertility in SCI patients and to discuss the best management strategies of these conditions as well as and future perspectives.

## 2. Male Sexual Dysfunction in SCI Patients

### 2.1. Ejaculatory Disorders

The ejaculation process consists of two different phases. Firstly, there is the emission phase, in which both the sympathetic and parasympathetic systems of the autonomic nervous system are involved [19,20]. During emission, the semen (glandular secretions) is mixed with the sperm and the resultant mixture is pushed through the internal segments of the urethra by smooth muscle–mediated movements while the bladder neck is closed. The parasympathetic preganglionic neurons are in the sacral parasympathetic nucleus, (S2–S4) whereas the sympathetic preganglionic neurons reside in the intermediolateral cell column and the central gray matter of the T12 to L2 spinal cord [21]. The sympathetic system sends axons into the hypogastric nerve and into the sympathetic trunk, while the parasympathetic system sends axons into the pelvic nerve. After emission, there is the expulsion phase, which consists of the expulsion of the semen through the external urethral orifice thanks to several coordinated and rhythmic contractions of several muscles, such as the bulbospongiosus (which is the most important muscle in the ejaculatory expulsion phase and is primarily involved in the firm erection of the glans penis), the ischiocavernosus muscles, the striated perineal muscle, and the external urethral sphincter [22]. The ischiocavernosus, external urethral sphincter, and bulbospongiosus stimulations are provided by several motor neurons located in Onuf’s nucleus, in the sacral segments, and in the pudendal nerve [22,23]. Therefore, if a patient has a lesion in the somatic center located in S2–S4, from which the pudendal nerve fibers supply the bulbospongiosus and ischiocavernosus muscles and the muscles of the pelvic floor, the lesion affects the second phase of ejaculation, and the ejaculation is not projectile. On the other hand, if there is a lesion in the sympathetic center (T11–L2), then the first phase of the process is affected [24]. Even so, most patients affected by SCI require assistance to ejaculate, and Kathiresan et al. [25] showed that ejaculation can occur by masturbation. Specifically, among SCI patients, men with incomplete injuries can ejaculate by masturbation [25].

### 2.2. Erectile Dysfunction

ED is defined as “the persistent inability to attain and maintain an erection sufficient to permit satisfactory sexual performance” [2]. Neurotransmission plays a key role in the erectile response. The main neurotransmitters involved in erectile response are acetylcholine, noradrenaline, and adrenaline. Pelvic nerves, hypogastric nerves, and pudendal nerves cooperate in the erectile response [21,22]. Specifically, an erection is produced by the dilatation of the arterioles (due to the acetylcholine relapse from the pelvic nerves that inhibits the noradrenaline relapse from the hypogastric nerves of the corpora cavernosa) that supply the erectile tissue of the corpora cavernosa [26]. As a result, the erectile tissue fills with blood, the erectile tissue veins become compressed, and the venous outflow decreases. In addition, acetylcholine triggers the release of nitric oxide (NO), a powerful smooth-muscle relaxant, and vasoactive intestinal polypeptide (VIP). The NO activates a molecular pattern of cyclic guanosine monophosphate (cGMP) that increases in the corpora cavernosa, relaxing the smooth muscle [26]. Conversely, phosphodiesterase 5, which converts cGMP into GMP, can be inhibited to promote and prolong the erection. A lesion at different levels of the spinal cord can impair the above mechanism, resulting in ED [26]. Actually, in 95% of men with complete SCI, a penile erection can occur, but it remains insufficiently rigid or sustained during sexual intercourse [26].

## 3. Infertility in SCI Patients

The term infertility describes “the inability of a sexually active and non-contraceptive couple to achieve a spontaneous pregnancy within 1 year” [27,28]. Infertility may be classified into two groups: primary infertility, which refers to a couple that has never had a child and cannot achieve pregnancy within 1 year, and secondary infertility, which refers to a couple that has had at least one pregnancy in the past [29]. Moreover, infertility can be permanent and irreversible, as in the case of male azoospermia (male sterility), or reversible, when the probability of spontaneous pregnancy is strongly reduced (subfertility) [29,30]. Traditionally, infertility has been associated with the female partner, held as responsible for the failure in conception. Specifically, 50% of cases are associated with male factors, possibly due to several causes (as shown in Table 1). In 30–40% of cases, no detectable factors can be individuated to explain the alterations in semen parameters. Finally, 20–30% of infertility is not explained by any sperm-quality alterations, which defines unexplained infertility [31]. It is believed that many unexplained cases of infertility are associated with sperm DNA damage and defective repair. Indeed, oxidative stress is the result of complex mechanisms due to biological factors (age, ejaculation frequency, varicocele, infection), lifestyle factors (smoking, obesity), and environmental factors (heat, other forms of electromagnetic radiation, toxins) that can impair spermatogenesis, triggering spontaneous mutations in the progenitors of spermatozoa [32]. Specifically, advanced paternal age represents a risk factor associated with the progressive increase in the prevalence of infertility factors in males [33]. The investigation of male infertility is easy and cheap, it is always recommended in the case of couples coming for infertility consultation, and it must be performed at the beginning of the diagnostic process.

### Genetic and Epigenetic Involvement

In recent years, the modern literature has offered few genetic studies on the infertile male with SCI. Indeed, there are no genome-wide association studies (GWAS). One recent study by Shi et al. [34] used a rat model with RNA sequencing to identify several immune genes, cytokines, and ion-channel genes that play a crucial role in SCI pathways. This represents a starting point for evaluating new genetic patterns involved in human infertility due to SCI. It is necessary to consider that the paucity of GWAS is due to the isolated nature of the event in contrast to cancer, which is associated with heritable somatic genes to study. However, a genetic reason may explain how SCI impacts germline DNA and the reasons why the presentation and severity of the infertility in men with SCI occur in a broad spectrum. Furthermore, the study of epigenetic focuses must be evaluated [7]. It is common knowledge that spermatogenesis, fertilization, and early embryogenesis are affected by DNA methylation and histone modification [7]. Recently, Jenkins et al. [35] studied the differences in DNA methylation in two groups of non-SCI men with comparable semen analysis but vastly different fecundity. Moreover, smoking is a risk factor for impaired fertility [2]. Indeed, smokers can present impaired semen quality compared to non-smokers, and DNA methylation status can change widely among the two groups [7]. SCI and smoking increase inflammatory markers and reactive species of oxygen (ROS) [7]. It is intuitive that inflammation can impact the sperm’s epigenetic programming in SCI men compared to non-SCI controls. Nevertheless, this area is still unexplored. However, it could represent a forward-looking point of interest for building new assisted reproductive technology.

## 4. Diagnosis

The assessment of ED in SCI patients relies on sequential steps: physical examination, clinical testing, and the results of specific questionnaires [2,36,37]. The physical examination comprises the evaluation of pelvic sensation and of the urogenital reflexes [37]. The four reflexes examined are the bulbocavernosus reflex (pertaining to nerves S2–S3), the bulb anal reflex (nerves S3–S4), the external anal-sphincter reflex (nerves S3–S4), and the cutaneous-anal reflex (nerves S4–S5) [37]. The presence of these reflexes implies the integrity of the sacral pathway. Thus, the reflex of erectile function is maintained. Moreover, a dermal sensation evaluation of the genitals and perineum is required to ensure the intactness of the T10–S5 segments [37]. Clinical testing comprises nocturnal penile tumescence (NPT) evaluation, measurement of skin potentials, measurement of the bulbocavernosus reflex, and sometimes urodynamic testing; unfortunately, these tests are not commonly performed in clinical practice [36,37]. Specifically, urodynamic testing can be applied to investigate the autonomic dysreflexia (AD) that can occur with or after SCI [38,39]. Finally, the most widely used instrument in SCI patients with ED is the International Index of Erectile Function (IIEF) [9]. The IIEF helps to understand the specific details of the erectile problems and to characterize the lesion itself [9]. Nevertheless, the evaluation of the medical and sexual history of SCI patients is required to guarantee optimal management of the disease according to an accurate physical examination. Indeed, the physician should characterize the impact of the ED on the patient’s daily life, the urgency of treatment, and the psychosocial factors related to the ED that may mutually influence or which may be caused by the existing ED itself. In conclusion, the physician should investigate the factors that may interfere with sexual function, such as comorbidities, drug therapies, and lifestyle habits.

## 5. Management of ED in SCI

### 5.1. PDE5Is

When the spinal cord is damaged, multiple medical therapies can be employed to manage ED in SCI patients, such as PDE5Is, ICI, VEDs, nutraceutical products, and erectogenic-urethral suppositories, as reported in Table 2 [37,40,41]. The main predictor of success with PDE5 inhibitors is the level of the spinal cord lesion [37]. If the penile vasculature is not involved and at least one nerve pathway is preserved (i.e., either the sacral or thoraco-lumbar pathway), PDE5Is such as sildenafil, tadalafil, and vardenafil, represent first-line therapies for ED in these patients [37,42]. Conversely, patients with lower-level of SCI do not benefit from this treatment. A reason for this phenomenon may be related to the drugs’ mechanism of action: specifically, PDE5Is sustain cAMP and cGMP intracellular levels, maintaining stable (but not increasing) NO levels. Both Afferi et al. [37] and Fenstermaker et al. [42] reported several treatment-emergent adverse effects (TEAEs) in relation to the use of PDE-5Is in SCI patients. The most common side effects associated with sildenafil are headache (16%), followed by flushing, dyspepsia, fatigue, and orthostatic hypotension (already present in these patients); but the systolic blood pressure (SBP) results remain steady [37,42]. The explanation of this phenomenon may be that PDE5Is cause a reduction in peripheral vascular tone, and thus, the heart rate (HR) increases in a compensatory manner in order to maintain cardiac output. Tadalafil-related adverse effects or vardenafil-related adverse effects in SCI patients are similar to those of sildenafil, both in terms of type and severity. In conclusion, PDE5Is are commonly contraindicated in patients using nitrate medication, which is frequently the case in SCI patients suffering from autonomic dysreflexia.

### 5.2. Intracavernosal Injection

The use of ICI with either papaverine, phentolamine, or PGE1, or a combination of these medications, is considered an alternative for PDE5I failure or as a first-line treatment in case of contraindications to PDE5Is [37]. Specifically, SCI patients with a lower lesion level are more likely to respond to ICI than to oral pharmacotherapy [37]. The major concern of ICI use is that it has been associated with a high incidence of TEAEs, principally priapism and injection site pain (for patients which retain tactile sensations) [37]. Moreover, SCI patients may complain of penile bruising and swelling and penile plaque formation at the injection site. In addition to TEAEs, the high cost of the compounds and the desire for a permanent solution cause the drop-out of patients during the first year of treatment [37,42].

### 5.3. Vacuum Erection Devices

VED treatments consist of the application of a cylinder-shaped tool that is put around the base of the flaccid penis [37]. With the help of a pump, a vacuum is created, which promotes an increase in cavernous blood flow, thereby causing penile erection [37]. The venous outflow is avoided due to the application of a constriction ring at the base of the penis; thus, the erection is maintained.

### 5.4. Penile Prosthesis

When medical therapies are ineffective, surgery represents the salvage point. Fenstermaker et al. [42] distinguished penile prostheses of two types: inflatable and malleable [43]. Malleable prostheses are made of semi-rigid cylinders that can be bent upward during sexual intercourse. The disadvantages of malleable prostheses include their constant rigidity, which makes them uncomfortable and can cause social embarrassment. Inflatable penile prostheses (IPP) consist of two cylinders, a reservoir balloon, and a pump which is placed within the scrotum and allows fluid transfer from the balloon to the cylinder chambers when an erection is desired. This device has the advantage of providing rigidity when desired but offers better flaccidity when deflated. However, IPP is not devoid of complications, including hematoma, early and late infections, and technical problems (i.e., defects in the implants such as the rupture of the connecting tubes or distal cylinder erosion, cylinder aneurysm, extrusion, self-inflation, pump migration, and balloon displacement). Importantly, for tetraplegic men with poor hand dexterity, activating and deactivating the prosthesis may represent a concern. Verze et al. [44] investigated the role of semi-rigid penile prostheses (SRPP) and two-piece IPPs as a valid alternative option in patients with impaired hand dexterity due to neurological problems. Actually, the market is opening to these new devices, and it is foreseeable that future studies could focus their attention on new PP devices committed to reducing tetraplegic patient discomfort and increasing their independence. A systematic review performed by Pang et al. [45] confirmed that PP represents an option for SCI patients for the management of end-stage ED or urinary function, but due to the rate of complications, preoperative patient counseling is mandatory.

## 6. Management of Ejaculatory Disorders and Infertility in SCI

### 6.1. Penile Vibratory Stimulation

PVS is the first-line therapy for the treatment of patients in which all spinal cord components (both the sympathetic and the parasympathetic mechanisms) of the ejaculation reflex and the dorsal nerve of the penis are intact. This patient’s level of injury must be T10 or rostral [46]. There are two FDA-approved devices for PVS treatment, FertiCare (Multicept, Denmark) and the Viberect (Reflexonic, MD, USA). The treatment consists of vibration applied to the penis, usually on the dorsum or on the frenulum, for up to 10 min. The treatment can be intermittent or continued until antegrade ejaculation occurs. Sometimes it is possible to use a second vibrator or combination therapy, which also involves electrical stimulation if the single-vibrator technique fails [47]. Since patients suffering from SCI have a high risk of retrograde ejaculation, the bladder must be prepared. Therefore, a buffer solution can be installed in the bladder and a catheter is used to achieve the sperm [48]. Autonomic dysreflexia is a condition that can occur in patients with spinal cord injuries above the level of T6. In such patients, some precautions must be considered. Due to the high level of the injury, the sympathetic inhibitory signal is compromised, and a noxious stimulus can result in an extreme sympathetic response which can lead to hypertension, peripheral vasoconstriction, diaphoresis, and hemodynamic instability. Brindley et al. [49], in 1981, treated 81 patients affected by paraplegia for more than 6 months. He obtained an ejaculation rate of 59% (48 out of 81) using a PVS. Brindley also allowed patients to inseminate their wives at home using their semen and a 5 cc syringe. He obtained 11 pregnancies using this protocol. Dahlberg et al. [50] also reported, in a survey conducted in the Craig Hospital, a good ejaculation rate (28 out of 57) using a home vibratory-stimulation program, with eight pregnancies detected. Ohl et al. [51] studied 11 patients affected by spinal cord injuries who had undergone PVS and EEJ in random order to detect any superiority in the two treatments. There was a slight advantage in sperm quality, in terms of the egg penetration rate, and the sensation of pain during the treatment for the branch treated with PVS. In 2010, Brackett NL et al. [52] conducted a retrospective study of data from 1991 to 2009, showing a success rate of 86% using PVS in men suffering from SCI with a level of injury at T10 or rostral and a 15% success rate for those with the level of injury above T11. Ibrahim et al. [53] advised using PVS as first-line therapy in the treatment of an ejaculatory disorder since it is non-invasive, cost-effective, safe, and usually simple to use. Moreover, in 2017 Sinha V et al. [54] showed that even though no significant improvement was found in terms of sperm mobility, the PVS technique allows a lower chance of retrograde ejaculation than the EEJ method. Fenstermaker et al. [42] suggested improving the safety of PVS by using a premedication with topical nitroglycerine paste or sublingual nifedipine.

### 6.2. Electro-Ejaculation

EEJ was firstly described by Gunn, a veterinary student, who performed the treatment on sheep in 1930 [55]. Then, 50 years later in 1980, Seager et al. [56,57] adapted the technique to use in humans. EEJ consists of the electrical stimulation of the nerves and smooth muscle of the pelvic muscles, as well as the prostate and seminal vesicles, through the rectum in patients affected by a complete SCI. In most patients suffering from a complete SCI, anesthesia is not requested except for cases with preserved pelvic sensation, in which conscious sedation or general anesthesia may be used. Usually, EEJ is considered second-line therapy for patients who failed with PVS. The change in retrograde ejaculation is higher for EEJ than for PVS; therefore, if an antegrade ejaculation is not obtained following the electrostimulation, a retrograde ejaculation may be suspected, and a catheter can be placed to collect semen from the bladder [53,58]. However, electrostimulation can be performed using a continuous or an interrupted method. The latter appears to be more valid for the collection of semen from the antegrade fraction [52]. For patients affected by an SCI at T6 or rostral, the blood-rising risk is higher, and premedication with nifedipine is required. Nevertheless, it is easier to manage since the stimulating current can be switched off [49]. Brackett et al. [52] conducted a retrospective study of 500 males between 1991 and 2009 where EEJ was performed in cases of failed PVS. They performed an EEJ 953 times in 210 patients with an ejaculation rate of 91.9% (193 patients out of 210) without any complications. Several side effects were described by several authors, such as Brindley et al. [49], whose 1981 study reported 256 EEJ in 89 males, with ejaculation rates of 64.1% (163 out of 256) for antegrade ejaculations and 17.1% for retrograde ejaculations (44 out of 256). They described several minor side effects such as contractions of striated muscles, after-effects of spasms, micturition, erections, rises in blood pressure, and contractions of the dartos muscle. Because potential complications also include rectal erythema or bleeding, most of the authors performed a rectal evaluation before starting the treatment [59]. Ohl et al. [51] worked on 48 patients, showing an ejaculation rate of 71% with no difference regarding age or the time duration between the procedure and the spinal injury. The authors achieved ejaculation in 60% of the males affected by cervical injury, while a higher rate was obtained in patients with thoracic spinal lesions.

### 6.3. Surgery

Several techniques for sperm retrieval have been described in the treatment of azoospermia, such as testicular fine-needle aspiration (TESA), percutaneous testicular biopsy, testicular sperm extraction (TESE), and microdissection testicular sperm extraction (Micro-TESE) [60]. Moreover, other surgical techniques, such as surgical epididymal sperm aspiration (MESA) and percutaneous epididymal sperm aspiration (PESA), are particularly useful in the treatment of obstructive azoospermia [60]. In 2015, Ibrahim et al. [53], noticing that sperm yields are usually low when retrieved surgically, suggested that surgical sperm retrieval should be considered as the last option in the treatment of patients with SCI. Otherwise, Iwahata et al. [61], one year later, suggested that spermatogenesis could worsen over time in SCI patients, and the discriminating factor in the spermatogenesis process in such patients could be their age at the time of the SCI. Moreover, the length of time from the SCI to surgery and the correlation with the sperm retrieval rate (SRR) were also evaluated, showing a trend of lower SRR in cases with a longer length of time since the SCI.

### 6.4. Other Treatments

Historically, several treatment options to achieve ejaculation in SCI patients have been studied, including subcutaneous physostigmine, intrathecal neostigmine, direct stimulation of the hypogastric nerve, and prostate massage. The rates of ejaculation obtained for these techniques were encouraging. Indeed, subcutaneous physostigmine ensured an ejaculation rate of 100% in patients affected by quadriplegia, of 33% in patients with a lesion at T1 ± T10 level, and of 0% in patients with a lesion at T11 ± L2 level [62]. Le Chapelain et al. [17] retrospectively analyzed 39 SCI patients who underwent PVS, EEJ and/or subcutaneous physostigmine administration (18 vs. 19 vs. 9 patients, respectively, considering 33 patients underwent a single technique for inducing ejaculation, 5 underwent two techniques, and 1 underwent all three techniques). The ejaculatory rate was 66% vs. 68% vs. 66%, respectively, according to the treatments choices. Moreover, Le Chapelain et al. [17] confirmed the influence of lesion level on the ejaculatory response. Indeed, among the SCI patients enrolled, the highest rates of ejaculatory response were detected in tetraplegic men (86% vs. 83% vs. 100%, according to the previously mentioned treatments choices, respectively). Instead, intrathecal neostigmine achieved a success rate of 59.7% for ejaculation [63]. Direct stimulation of the hypogastric nerve reached a 100% success rate for ejaculation [64]. Conversely, prostate massage guaranteed an ejaculation rate of 31.9%, according to Arafa’s analysis [65]. The concerns about these techniques (excluding prostate massage) were represented by the side effects, the invasiveness of the procedures, and their cost-effectiveness. As a result, they were not scheduled as first-line therapies Table 3.

### 6.5. Future Perspectives

The satisfactory treatment of this condition still remains a challenging point in the management of SCI. Stem-cell therapy and tissue engineering strategies played emerging roles in the management of SCI during the last decades [67]. Indeed, recent evidence from pre-clinical trials for SCI repair studied the implantation effects of a collagen scaffold made from bovine aponeurosis (NeuroRegen scaffolds) together with autologous human bone-marrow mononuclear cells (BMMCs) or allogeneic mesenchymal stem cells (MSCs) in animal models [67]. This system could be used to recover or improve sensory and autonomic nervous functions such as defecation sensation, physiological erections, sweating, and superficial or deep sensations in some SCI patients [67]. However, no motor function recovery was observed during the three-year clinical study. Thus, new clinical studies are largely required.

## 7. Conclusions

SCI is a relevant medical and social problem. Treatment options for both male sexual dysfunction and for infertility impairment must be tailored to patients’ individual needs and consider the clinical history of the SCI in these patients (specifically, age at SCI, time post-SCI, SCI level, and SCI completeness), their comorbidities, and the relevant available drugs. Despite multiple on-going trials, the bridge between preclinical experiments and clinical practice is still missing but represents a hope for the nearest future.

## Figures and Tables

**Table 1:** Common causes of SCI in general population according to World Health Organization (WHO) analysis.

Driving Incidents	Automobile Crashes
	Motorcycle Crashes
Falls	
**Acts of violence**	Gunshots wounds
	Self-harm
**Medical and surgical complications**	Inflammation of the spinal cord
	Arthritis
	Osteoporosis
	Cancer
**Sports and recreation injuries**	Athletic activities (e.g., impact sports or diving in shallow water)

**Table 2:** Main treatments related to the management of ED in SCI patients.

Treatment	Method of Administration	Indication	Advantages	Disadvantages
PDE5Is	Oral	First-line therapy	Oral therapy, steady blood pressure, rapid administration	Headache, flushing, dyspepsia, fatigue, orthostatic hypotension
Papaverine, Phentolamine, or PGE1	Intracavernosal injection	Alternative for PDE5I-failure or as a first-line treatment in case of contraindications to PDE5Is	Rapid administration	Priapism, injection site pain, penile bruising, swelling, penile plaque formation, high costs
Vacuum Erection Devices	Topical	-	Non-invasiveness, temporary effect	-
Penile Prosthesis	Surgical	End-stage ED or urinary function	Curative intent	Hematoma, early and late infections, technical problems
Inflatable prosthesis			Desired rigidity	-
Malleable prosthesis			-	Constant rigidity

Abbreviations: phosphodiesterase-5 inhibitors, PDE5Is; erectile dysfunction, ED.

**Table 3:** Main findings relative to the management of male ejaculatory disorders in SCI patients. The main outcome was the ejaculation rate obtained in SCI patients using several different approaches: PVS, EEJ, subcutaneous physostigmine, intrathecal neostigmine, the direct stimulation of the hypogastric nerve, and prostate massage.

Author	Study Population	Treatment	Ejaculation Rate Obtained (%)	Advantages	Disadvantages
**Ohl DA et al.** [48]	34 patients	PVS	65%	Non-invasiveness Safety Cost-effectiveness Usability Success rate	Hemodynamic instability Diaphoresis Autonomic dysreflexia Edema or abrasion of penile skin Retrograde ejaculation
**Brindley et al.** [49]	81 patients	PVS	59%		
**Dahlberg et al.** [50]	57 patients	PVS	49.1%		
**Brackett NL et al.** [52]	500 patients	PVS	86%		
**Ohl et al.** [66]	48 patients	EEJ	71%	Success rate Applicable in PVS-failure patients	Invasiveness Hemodynamic instability Autonomic dysreflexia Rectal and anal burns
**Guttman and Walsh** [63]	70 patients	Prostigmin	59.7%	Reversible Non-invasiveness	Parasympathetic side-effects Hemodynamic instability Requires ECG monitoring
**Brindley et al.** [64]	7 patients	Hypogastric plexus stimulators	100%	Success rate Non-invasiveness	Hypogastric plexus lesions
**Arafa MM** [65]	69 patients	Prostatic massage	31.9%	Non-invasiveness Safety Cost-effectiveness Usability	Patient discomfort

Abbreviations: penile vibratory stimulation, PVS; electro-ejaculation, EEJ; spinal cord injury, SCI.

## Data Availability

The data presented in this study are openly available.
